# Elucidating Mechanisms of Hypomorphic WDR19-Related Kidney Failure

**DOI:** 10.1016/j.ekir.2025.07.019

**Published:** 2025-07-24

**Authors:** Omer Shlomovitz, Yam Ben-Haim, Netanel Eisenstein, Leah Armon, Igor Grinberg, Sylvie Polak-Charcon, Danit Atias-Varon, Guy Chowers, Dror Ben-Ruby, Achia Urbach, Asaf Vivante

**Affiliations:** 1 Department of Pediatrics B, Edmond and Lily Safra Children’s Hospital, Sheba Medical Center, Tel-Hashomer, Ramat Gan, Israel; 2 Gray Faculty of Medical and Health Sciences, Tel-Aviv University, Israel; 3 Genetic Kidney Diseases Research Laboratory, Sheba Medical Center, Israel; 4 The Mina and Everard Goodman Faculty of Life Sciences, Bar-Ilan University, Ramat-Gan, Israel; 5 Sheba Medical Center, Institute of Pathology, Tel-Hashomer, Ramat Gan, Israel; 6 Pediatric Nephrology Unit, Edmond and Lily Safra Children’s Hospital, Sheba Medical Center, Tel-Hashomer, Ramat Gan, Israel

**Keywords:** ciliopathies, kidney organoids, WDR19

## Abstract

**Introduction:**

Variants in the *WDR19* gene, a crucial component of the intraflagellar transport (IFT) complex A, are associated with renal-cystic ciliopathies, a prevalent cause of renal failure of genetic origin. In the Arab Druze population, a *WDR19* pathogenic missense variant (c.878G>A; p.Cys293Tyr, termed *WDR19:C.878G>A*) is the most common genetic cause of kidney failure manifesting as adult-onset, typically nonsyndromic chronic kidney disease (CKD). The underlying pathogenesis of this condition remains unclear.

**Methods:**

We used CRISPR-Cas9 to induce patient-specific hypomorphic and loss-of-function (LoF) variants in human embryonic stem cells (hESCs), in addition to using patient-derived induced pluripotent stem cells (iPSCs) for differentiation into kidney organoids. Organoids were assessed by using immunofluorescence, electron microscopy, RNA-sequencing, and pathway analysis to elucidate the effects of these pathogenic variants on kidney development and ciliopathy characteristics.

**Results:**

The *WDR19* hypomorphic variant impairs nephron development, causing delayed kidney organoid differentiation from early stages, cystogenesis, and structural abnormalities in both tubular and glomerular structures. Mutant organoids displayed reduced ciliation and shortened cilia. Both mutated organoids exhibited Sonic hedgehog dysregulation, where the pathway was upregulated in the presence of severe LoF variant and significantly reduced ciliation. Elevated sonic hedgehog (Shh) signaling was associated with significant downregulation of fibroblast growth factor (FGF) 8 (FGF8) and transcriptomic alterations in associated pathways, suggesting an inverse pathways relationship during kidney organoid development.

**Conclusion:**

Our study validates the pathogenic role of the *WDR19* hypomorphic variant in adult-onset renal failure and highlights how hypomorphic pathogenic variants disrupt kidney development. These findings underscore the critical role of cilia in renal development, offering insight into the mechanisms of ciliopathies.

## Graphical abstract

**Figure:**
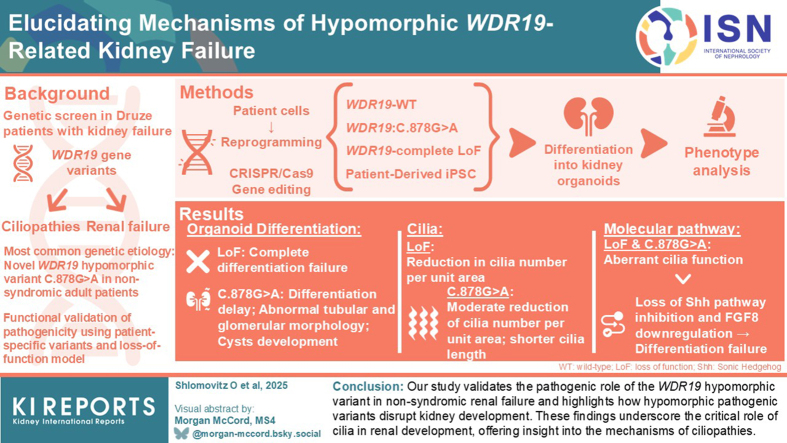

Genetic variants are increasingly recognized as key contributors to the pathogenesis of end-stage kidney disease (ESKD). Understanding the genetic basis of kidney disease is crucial for improving diagnosis and management,^1^ particularly in cases where clinical presentations are nonspecific. Ciliopathies are the most common cause of CKD of genetic origin.^2^^,^^3^ Unlike the more recognizable syndromic ciliopathies that manifest in early childhood with multiorgan involvement, adult-onset ciliopathies can be misdiagnosed or remain undetected until the presentation of kidney failure. In many cases, adult patients with ciliopathies may exhibit CKD of unknown etiology, with the absence of extrarenal symptoms, further complicating the diagnostic process.^4^^,^^5^ This can lead to delays in appropriate genetic testing and treatment.

In our previous study,^6^ we identified the homozygous NM_025132.4 *WDR19*:c.878G>A p.Cys293Tyr pathogenic variant in the *WDR19* gene as the most common cause of CKD of genetic origin in Druze individuals. The variant typically leads to nonsyndromic ESKD between the ages of 20 and 40 years (Supplementary Figure S1 and Table S1). Renal biopsies from affected individuals revealed global sclerosis, severe interstitial fibrosis, and, in some cases, IgA depositions. Notably, many Druze patients were initially misdiagnosed with glomerular diseases, such as focal segmental glomerulosclerosis or IgA nephropathy, highlighting the challenges in accurately diagnosing and classifying ciliopathy-related adult-onset CKD. Given the lack of clear ciliopathic characteristics in our patients, who present with adult-onset ESKD, we opted to validate pathogenicity of this population-specific *WDR19* variant.

*WDR19* encodes IFT144, a component of the IFT complex A, crucial for intraciliary transport.^7^ The cilium is extended and maintained through particle transport along the axoneme, mediated by the IFT system. Anterograde trafficking, driven by IFT-B and Kinesin-2, is essential for cilia formation; whereas retrograde transport, dependent on IFT-A and Dynein-2 (i.e., Dync2h1), is crucial for maintaining function. Disruption of retrograde transport typically results in shortened, malfunctioning cilia.^8^, ^9^, ^10^, ^11^ Previously, we demonstrated that *WDR19* is abundantly expressed in human embryonic renal tissue and is present in both tubular and glomerular cells, specifically in parietal epithelial cells in adult kidneys.^6^

Pathogenic variants in *WDR19*, first reported by Bredrup *et al.*,^12^ are associated with ciliopathies and have been described in a limited number of case reports.^13^, ^14^, ^15^ These reports predominantly describe children with severe multiorgan syndromes, displaying extrarenal manifestations such as skeletal disorders, liver disease, and ophthalmologic involvement. These patients often progress to ESKD early in life, sometimes even during infancy. The renal phenotypes linked to these *WDR19* variants are not well-characterized, showing diverse histological abnormalities, including interstitial fibrosis, atrophic tubules, glomerular sclerosis, glomerular cysts, and rarely, evidence of IgA deposits, which may mimic immune complex–mediated glomerulopathy, complicating the understanding of disease etiology.^13^^,^^15^ Proteinuria is occasionally reported.^16^

The ciliary-associated Shh pathway is key to regulating cell differentiation, tissue patterning, and embryonic development. Shh components, such as the smoothened (i.e., SMO) protein and the Gli transcription factors, are localized to the cilium. Notably, recent studies have shown that cilia play a pivotal role in mediating the switch from Shh pathway activation to repression during kidney organoid differentiation.^17^ When Shh binds to its receptor Patched (i.e., PTCH1), the inhibition on the SMO protein is released, allowing it to move into the cilium and activate the Gli transcription factors, which then translocate to the nucleus to regulate target gene expression.^18^, ^19^, ^20^ In the absence of Shh ligand, Gli transcription factors are processed into their repressor forms, particularly Gli3R, which maintains the inactivity of Shh target genes. The role of IFT proteins in Shh signaling is multifaceted, because of their crucial function in maintaining cilia structure, which is essential for Shh activity. Conversely, IFT is critical for the formation of Gli3R by facilitating the proper trafficking and proteolytic processing of Gli3 within the primary cilium. Thus, disruption of IFT proteins can compromise this repressive function, potentially resulting in Shh pathway overactivation.^21^ Despite progress in understanding IFT’s role as a regulator of the Shh pathway, the impact of *WDR19* variants on Shh signaling remains unclear, and it is uncertain whether hypomorphic and LoF variants have distinct effects on the pathway.^22^^,^^23^

In this study, we validated the pathogenicity of the hypomorphic *WDR19* (c.878G>A; p.Cys293Tyr) variant and investigated whether its effects extend beyond tubulointerstitial pathogenicity, potentially explaining the glomerular involvement and histological features observed in our patients and those described in the literature. Furthermore, we sought to gain a clearer understanding of the distinctions between hypomorphic and complete LoF variants within an IFT-A component, their impact on Shh signaling, and downstream transcriptomic changes.

## Methods

Approval for human subject research was obtained from the institutional review board of Sheba Medical Center (SMC-9929-22). Written informed consent was obtained from all participating patients.

### Cell Culture

iPSCs and hESCs were grown feeder-free on vitronectin- (Stemcell Technologies) coated plates in mTeSR1 growth medium (Stemcell Technologies) at 37 °C in a 5% CO_2_ atmosphere. Cells were passaged either with ReLeSR (Stemcell Technologies) or Accutase (Sigma) for single-cell passaging. The cells were treated with 10 mM ROCK1 inhibitor (Y27632 hydrochloride, Apexbio) overnight upon single-cell passaging.

### RNA Extraction and Real-Time Quantitative Polymerase Chain Reaction

Total RNA was extracted using Direct-zol RNA MiniPrep Kit (Zymo Research), followed by reverse transcription using iScript cDNA Synthesis Kit (BioRad). Real-time quantitative polymerase chain reaction was performed using PerfecTa SYBR Green FastMix (Quantabio). HPRT gene was used for normalization. The list of primers used are presented in Supplementary Table S2.

### Immunostaining

For immunofluorescent staining of cilia, ciliogenesis was promoted by starving kidney organoids of serum for 48 hours before fixation, using DMEM/F12 (Sartorius) without fetal bovine serum. For all types of staining, organoids were fixed with 4% paraformaldehyde for 40 minutes at room temperature and washed in phosphate-buffered saline. The cells were blocked in phosphate-buffered saline with 10% to 20% fetal bovine serum (Sigma), 0.02% glycine (Bio-Lab) and 0.1% to 0.3% Triton X-100 (Fisher Chemical) for 1 to 3 hours at room temperature, followed by incubation with primary antibodies (Supplementary Table S3) at 4 °C overnight. After washing the organoids 6 times for 10 minutes each, they were incubated with secondary antibodies (Supplementary Table SD) for 3 hours at room temperature. The nuclei were counterstained with DAP (Sigma) for 10 to 90 minutes at room temperature. Samples were washed 3 times with phosphate-buffered saline for 10 minutes each and then mounted with Fluoromount-G (Invitrogen). Immunostained samples were imaged by inverted Leica DMi8 scanning confocal microscope, driven by the LASX software (Leica Microsystems). For large areas, multiple images were acquired, and tiles were automatically merged into a single image with the LAS X software.

### Directed Differentiation to Kidney Organoids

The differentiation was according to the protocol by Takasato *et al.*^24^ Undifferentiated iPSCs and hESCs were maintained in feeder-free conditions on Vitronectin- (Stemcell Technologies) coated 6-well plates in mTeSR1 medium (Stemcell Technologies). One day before differentiation, the cells were dissociated with TrypLE Select (Thermo Scientific) and 1.4 × 10^4^ cells were plated per well of a 6-well plate on hESC-qualified Geltrex (Thermo Scientific) in 2 ml mTeSR1 medium supplemented with 10 μM Y-27632 dihydrochloride (ApexBio). The following morning, cells were treated with 8 μM CHIR99021 for 4 days, followed by FGF9 (200 ng/mL, R&D Systems) and heparin (1 μg/ml, Stemcell Technologies) for 3 days, during which the medium was changed every 1 to 2 days. On day 7, cells were dissociated with TrypLE Select, counted and 250,000 cells were transferred to a ThinCert 0.4 μm pore polyester membrane (Greiner Bio-One). Cells were treated with 5 μM CHIR99021 for 1 hour and then incubated in medium containing FGF9 (200 ng/ml) and heparin (1 μg/ml) for 5 days. The culture continued without added growth factors for up to 24 days, during which the medium was changed every 2 days. Each of the cell lines, *WDR19*-G>A, *WDR19*-LoF, and *WDR19*-iPSCs, were independently differentiated at least 3 times, representing 3 biological replicates for each cell line. In each biological replicate, between 10 and 25 separate organoids were generated, which were distributed across 2 to 5 ThinCerts, with 5 organoids per well. In specifically designated transwells. Forskolin (Cayman Chemical), an adenyl cyclase activator, was used to induce cyst formation.

### RNA-Sequencing

Libraries were prepared using the INCPM mRNAseq protocol, and 100 bp single reads were sequenced on a NovaSeq S1 (100 cycles), yielding approximately 56 million reads per sample. Poly-A/T stretches and Illumina adapters were trimmed using Cutadapt, with reads shorter than 30 bp discarded. The trimmed reads were mapped to the human reference genome GRCh38_p13 using STAR, and uniquely mapped reads were processed to remove duplicates with the PICARD MarkDuplicate tool. Gene expression levels were quantified using htseq-count, and differentially expressed genes were identified using DESeq2, with *P*-values adjusted for multiple testing via the Benjamini-Hochberg procedure. Quality assurance was ensured by achieving an approximately 91% read mapping rate, with approximately 74% of uniquely mapped reads aligning to exons. For each cell line, multiple replicates were analyzed: *WDR19*-WT with 5 replicates, *WDR19:C.878G>A* with 4 replicates, and *WDR19*-iPSCs with 3 replicates. Each sample consisted of RNA purified from 3 to 4 kidney organoids. Differentially expressed genes were filtered according to the following criteria: adjusted *P*-value < 0.05, log2FoldChange > 1, and base mean > 20 (Supplementary Tables S5 and S6).

### Statistical Analysis

For the quantification of cilia length and number per area, unpaired *t* tests were performed. For real-time polymerase chain reaction and Shh activity, band intensities of GLI3F and GLI3R were measured, and their ratios were analyzed using unpaired *t* tests. Real-time polymerase chain reaction and Western blot quantifications were based on sample sizes of *n* = 3 to 4 for each genotype, representing RNA or protein collected from organoids pooled from 3 separate wells. Statistical analyses were conducted with 2-tailed *P*-values, and significance was set at *P* < 0.05. Additional information on the methods are presented in the Supplementary Methods.

## Results

**Figure 1:**
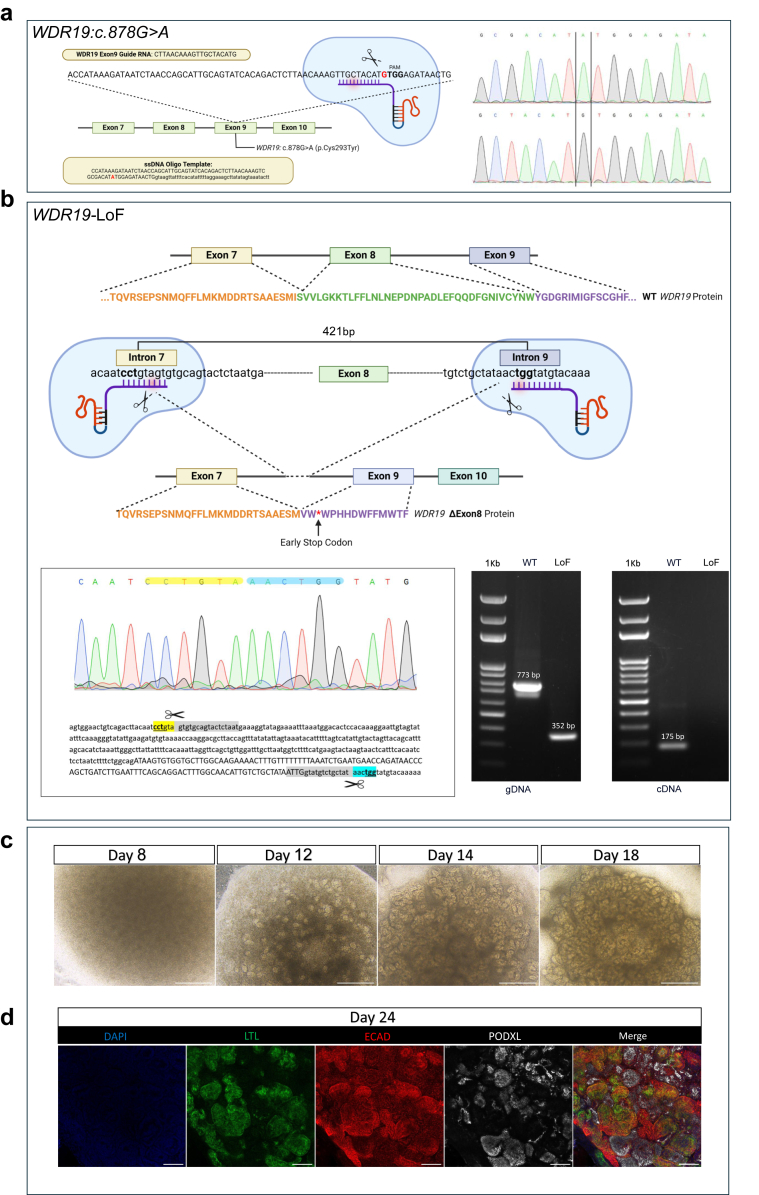
(a) Generation of CSES7 embryonic stem cell line with the induced *WDR19* c.878G>A pathogenic variant (*WDR19:C.878G>A*). Schematic representation of the plasmid-mediated CRISPR-Cas9 gene editing approach used in the current study. A plasmid containing guide RNA (gRNA) targeting the *WDR19* locus and Cas9 was introduced into cells. For homology-directed repair (HDR), a donor template, comprising the c.878G>A variant, adjacent silent variants, and flanking homology arms, was delivered to facilitate precise genome editing. On the right is the Sanger sequencing confirmation of the *WDR19:C.878G>A* induced clone, compared with the isogenic *WDR19*-WT control. (b) Generation of the CSES7 embryonic *WDR19* LoF embryonic stem cell line (*WDR19*-LoF). This CRISPR-Cas9 approach uses 2 gRNAs to create double-strand breaks in the intronic regions flanking Exon 8 of the *WDR19* gene. The deletion of Exon 8 leads to a frameshift, introducing an early stop codon and producing a truncated, non-functional *WDR19* protein. In the lower panel, the sequencing confirmation of the *WDR19*-ΔExon8 clone is shown, alongside electrophoresis results comparing *WDR19*-WT and the 432 bp shorter *WDR19*-ΔExon8 product. In addition, cDNA electrophoresis demonstrates no detectable product in the *WDR19*-ΔExon8 knockout line, confirming successful exon deletion and absence of full-length transcript. (c) WT kidney organoid generation with representative phase contrast images of the organoids on days 8, 12, 15, and 24 of differentiation; the latter is a mature kidney organoid. Scale bars: 500 μm. (d) Immunofluorescence confocal images of *WDR19*-WT line-derived kidney organoids on day 24 of differentiation. ECAD (red) marks the distal tubule, LTL (green) the proximal tubule, and PODXL (white) the glomeruli. Scale bars: 100 μm. WT, wild-type.

To investigate the effects of a patient-specific *WDR19:C.878G>A* hypomorphic and complete *WDR19*-LoF variants, we generated the following cell lines: CRISPR-Cas9-edited hESC line containing the c.878G>A; p.Cys293Tyr variant (herein *WDR19:C.878G>A*) (Figure 1a) and its isogenic wild-type (WT) control *WDR19*-WT, *WDR19:C.878G>A* patient-derived iPSCs (herein *WDR19*-iPSCs), and a cell line with LoF *WDR19* variant (herein *WDR19*-LoF) (Figure 1b). To minimize batch-specific effects, we repeat each morphological and immunofluorescence experiment at least 3 times, analyzing a minimum of 3 organoids per genotype in each experiment.

### The *WDR19* Gene is Essential for Human Kidney Development

**Figure 2:**
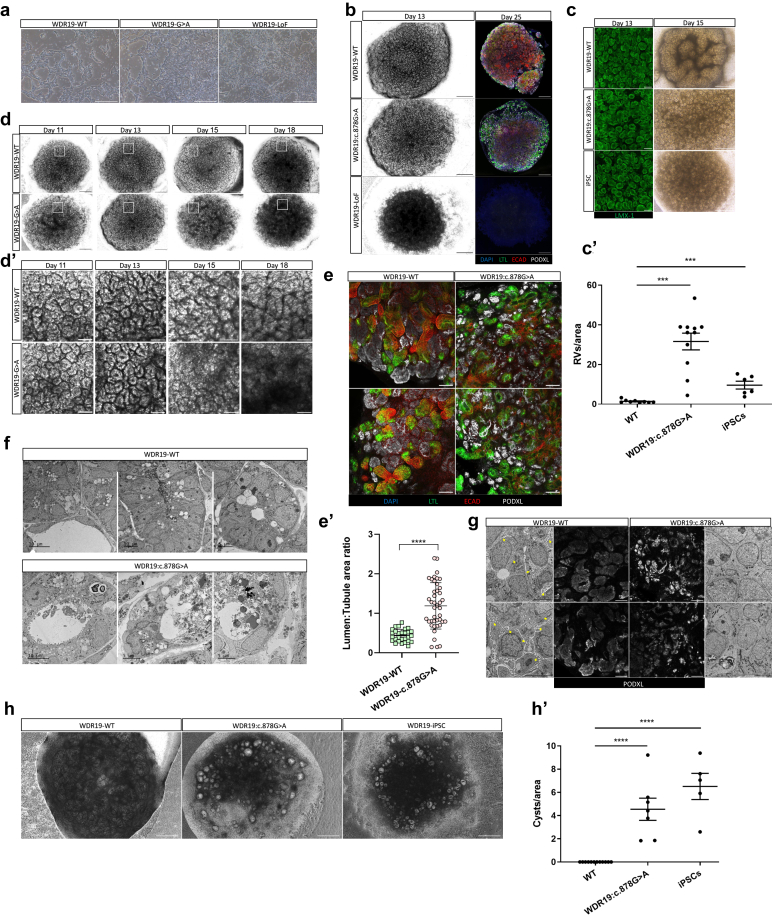
(a) Phase contrast images of the 3 human embryonic stem cell lines with identical genetic backgrounds—*WDR19*-WT, *WDR19:C.878G>A*, and *WDR19*-LoF—on day 4 of differentiation into kidney organoids at the monolayer stage. At this stage, the cells exhibit similar morphology. Scale bars: 500 μm. (b) Left panel: phase contrast representative images. of day 13, *WDR19*-WT and *WDR19:C.878G>A* organoids show*ing* proper differentiation, reaching the renal vesicle stage, whereas no such structures are observed in *WDR19*-LoF organoids. Right panel: day 25 immunofluorescence images display nephron compartments. A noticeable scarcity of nephron structures is seen in *WDR19*-LoF organoids compared with *WDR19*-WT and hypomorphic *WDR19:C.878G>A* organoids. Scale bars: 500 μm. (c) Representative images of kidney organoids derived from *WDR19*-WT, *WDR19:C.878G>A*, and *WDR19*-iPSCs on days 13 and 15. On day 13, all 3 lines show similar rounded luminal structures positive for LHX1 (green), marking renal vesicles. By day 15, *WDR19:C.878G>A* and *WDR19*-iPSC organoids retain rounded “doughnut-like” renal vesicles, whereas *WDR19*-WT organoids progress to a more tubular and convoluted morphology, indicating advanced differentiation. (c’) Quantification of renal vesicles (RVs) per area in kidney organoids derived from *WDR19*-WT, *WDR19:C.878G>A*, and *WDR19*-iPSCs. *WDR19:C.878G>A* organoids display a significantly higher number of renal vesicles compared with *WDR19*-WT. Data points represent individual measurements, and error bars indicate the mean ± SEM. ∗∗∗*P* < 0.001. For renal vesicle quantification, *WDR-G>A*: *n* = 11, *WDR-WT*: *n* = 7, iPSC: *n* = 6. Scale bars: 100 μm (right) and 500 μm (left). For more information, see Materials and Methods section. (d) Phase contrast images showing differentiation timeline from days 11 to 18. *WDR19:C.878G>A* organoids show a markedly less organized appearance and early collapse starting from day 15 of differentiation. (e) Immunofluorescence images of mature kidney organoids (day 25) derived from *WDR19*-WT and *WDR19:C.878G>A* lines. *WDR19:C.878G>A* organoids exhibit abnormal tubular structures, including dilated LTL-positive proximal tubules (yellow arrowheads) and lack of structural continuity between different nephron compartments. Scale bars: 100 μm. (e’). Tubule-to-lumen area ratio analysis based on immunostaining images demonstrates significantly tubular dilatation in *WDR19*:C.878G>A organoids relative to *WDR19*-WT. Data is presented as mean ± SD, ∗∗∗∗*P* < 0.0001.(f) Electron microscopy images revealing disrupted tubular morphology, dilated lumens, and accumulation of cellular debris in *WDR19:C.878G>A* organoids, further indicating abnormal nephron architecture compared to *WDR19*-WT. Scale bars: 5–10 μm. (g) Electron microscopy images of kidney organoids revealing foot process–like structures resembling podocyte pedicels in *WDR19*-WT organoids (yellow arrowheads), which are absent in *WDR19:C.878G>A* organoids. Scale bars: 5 nm. Middle: immunofluorescence images of PODXL staining showing that *WDR19:C.878G>A* organoids exhibit smaller, less uniformly rounded glomerular structures compared to *WDR19*-WT. Scale bars: 100 μm. (h) Cystogenesis in kidney organoids treated with forskolin (25 μM) from days 14 to 25 of differentiation. Representative images show cyst-like dilations in *WDR19:C.878G>A* and *WDR19*-iPSC derived organoids, whereas *WDR19*-WT organoids do not form cysts. (h’). Quantification of cysts per area indicates a significant increase in cyst formation in *WDR19:C.878G>A* and iPSCs compared with *WDR19*-WT organoids. Data are presented as mean ± SEM, ∗∗∗∗*P* < 0.0001. *WDR19*-WT: *n* = 12; *WDR19*G>A: *n* = 7; iPSC: *n* = 5. Scale bars: 500 μm. LoF, loss-of function; WT, wild-type.

To explore the effect of the *WDR19* variants on kidney development, we differentiated the WT and mutated cells into kidney organoids (Supplementary Figure S2). No morphological differences were observed among the *WDR19*-WT, *WDR19:C.878G>A*, and the *WDR19*-LoF up to day 8 of differentiation (Figure 2a and Supplementary Figure S3). However, beyond day 8, during the transition from intermediate mesoderm to distinct kidney-specific lineages, the *WDR19*-LoF cells failed to further differentiate. None of the *WDR19*-LoF kidney organoids developed early nephron structures, such as renal vesicles or later tubuloid structures, as seen by phase contrast microscopy (Figure 2b, left panel). Immunostaining for tubular markers, including the glomerular (PODXL), proximal (LTL) and distal (ECAD) tubules, revealed a marked lack of differentiation in the *WDR19*-LoF cells compared with the *WDR19*-WT cells (Figure 2b, right panel). This observation is consistent with the absence of homozygous *WDR19* LoF variants described in the literature, implying nonviability, and with the observation that bi-allelic LoF variants in mice result in prenatal lethality.^22^^,^^23^ Notably, in contrast to the *WDR19*-LoF line, the *WDR19:C.878G>A* shows a milder tubular differentiation failure (Figure 2b, right panel).

### *WDR19*-Mutated Kidney Organoids Recapitulate Ciliopathy Characteristics and Exhibit Multiple Abnormal Morphologies

On day 13 of differentiation, the 3 lines, *WDR19*-WT, *WDR19:C.878G>A*, and *WDR19*-iPSCs, exhibited highly similar morphologic appearances with rounded luminal structures positive for LHX1, an intermediate mesoderm marker indicating renal vesicles (Figure 2c, left panel). By day 15, the *WDR19*-WT organoids progressed to a more tubular and convoluted appearance, indicating a higher level of differentiation (Figure 2c, right panel; Supplementary Figure S4 and Figure 2c’). In contrast, the 2 mutated kidney organoids (*WDR19:C.878G>A* and *WDR19*-iPSCs) predominantly retained rounded, “doughnut-like” renal vesicle structures. Thus, morphologically, the *WDR19:C.878G>A* kidney organoids exhibited a delay in differentiation compared with WT organoids. Notably, the mutated organoid structures showed increased collapse and disorganization of nephron architecture during the later stages of differentiation, starting around day 15, compared with *WDR19*-WT cells (Figure 2d and 2d’). Mature kidney organoids (day 25) exhibited multiple dysmorphologies, including dilated LTL-positive (proximal tubule) structures, and lack of continuity between different organoid structures, such as LTL-positive proximal tubules, ECAD-positive distal tubules, and PODXL-positive glomerular structures (Figure 2e). The failure to form organized epithelial tubules was further supported by ZO1 staining—a tight junction marker—which revealed aberrant cellular localization in the diseased organoids (Supplementary Figure S5). In addition, representative qualitative images by electron microscopy revealed disrupted tubular morphology, dilated lumens, and luminal accumulation of cellular debris (Figure 2f). Frequently, in *WDR19*-WT organoids, foot process–like structures resembling podocyte pedicels were observed by electron microscopy (Figure 2g**,** yellow arrowheads), indicating the complexity of organoid-derived glomerular structures and proper development. However, these structures were absent in a thorough analysis of *WDR19:C.878G>A* organoids sections. Furthermore, PODXL-positive glomerular structures in *WDR19:C.878G>A* kidney organoids appeared smaller and less uniformly rounded (Figure 2g). These results suggest that even hypomorphic *WDR19* pathogenic variants impact kidney development at early stages. Given that nephronophthisis often presents with small corticomedullary kidney cysts, to further assess whether our model accurately recapitulates this ciliopathy phenotype, we applied Forskolin, an adenyl cyclase activator, to cultures from days 14 to 25 of differentiation to induce cyst formation. Both *WDR19:C.878G>A* mutant and iPSC-derived kidney organoids developed multiple cyst-like dilations in tubular structures, effectively mimicking a ciliopathy phenotype. In contrast, Forskolin treatment did not alter the structural morphology in *WDR19*-WT organoids (Figure 2h and h’).

### The *WDR19* Hypomorphic Variant Leads to a Reduced Number of Ciliated Cells With Shorter Cilia Whereas Nonsense Variant Leads to Severe Scarcity of Ciliated Cells

**Figure 3:**
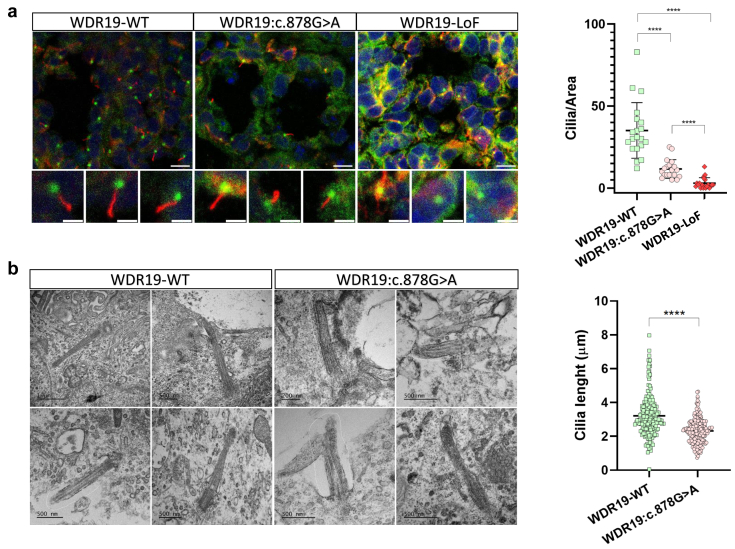
(a) Immunofluorescence staining of cilia using acetylated alpha-tubulin (red) and gamma-tubulin (green) as markers for axonemal structure and basal body, respectively. Both the *WDR19:C.878G>A* and *WDR19*-LoF cells exhibit significantly reduced cilia per unit area compared to wild-type cells (∗∗∗∗*P* < 0.0001), with *WDR19*-LoF showing the most severe reduction. Scale bars: 10 μm (upper panel) and 3 μm (lower panel). (b) Transmission electron microscopy images of primary cilia in WT and *WDR19:C.878G>A* cells. Cilia from *WDR19*-WT (left panel) appear normal, exhibiting typical structural features, whereas cilia from *WDR19:C.878G>A* mutant cells are shortened. Scale bars: 500 nm and 200 nm. The graph on the right quantifies cilia length based on *n* = 200 random measurements of acetylated alpha-tubulin positive cilia. Cells carrying the *WDR19:C.878G>A* variant exhibit significantly shorter cilia compared to WT cells (∗∗∗∗*P* < 0.0001). LoF, loss-of function; WT, wild-type.

Disrupted retrograde transport of ciliary proteins due to IFT-A complex deficiencies results in abnormal ciliary morphology, including shortened primary cilia with swollen tips.^25^ Accordingly, cilia in *WDR19*-KO mice were shorter or missing entirely.^26^ Thus, we analyzed the effect of the *WDR19:C.878G>A* and *WDR19*-LoF variants on the cilia. Primary cilia were clearly identified in the tubular epithelium of the WT kidney organoids; however, the *WDR19:C.878G>A* organoids exhibited a significant reduction in ciliary count per unit area, compared with the WT. This reduction was more pronounced in *WDR19*-LoF organoids (Figure 3a). Examination of ciliary length based on the immunostaining and transmission electron microscopy images revealed that cilia from *WDR19:C.878G>A* mutant cells were significantly shortened compared with those observed in *WDR19*-WT cells (Figure 3 b and c). The reduction in both cilia length and number suggests that hypomorphic *WDR19* variant impairs ciliogenesis or ciliary maintenance.

### Both *WDR19* Hypomorphic Variant and Complete *WDR19* LoF led to Increase in Activation of the Shh Pathway

**Figure 4:**
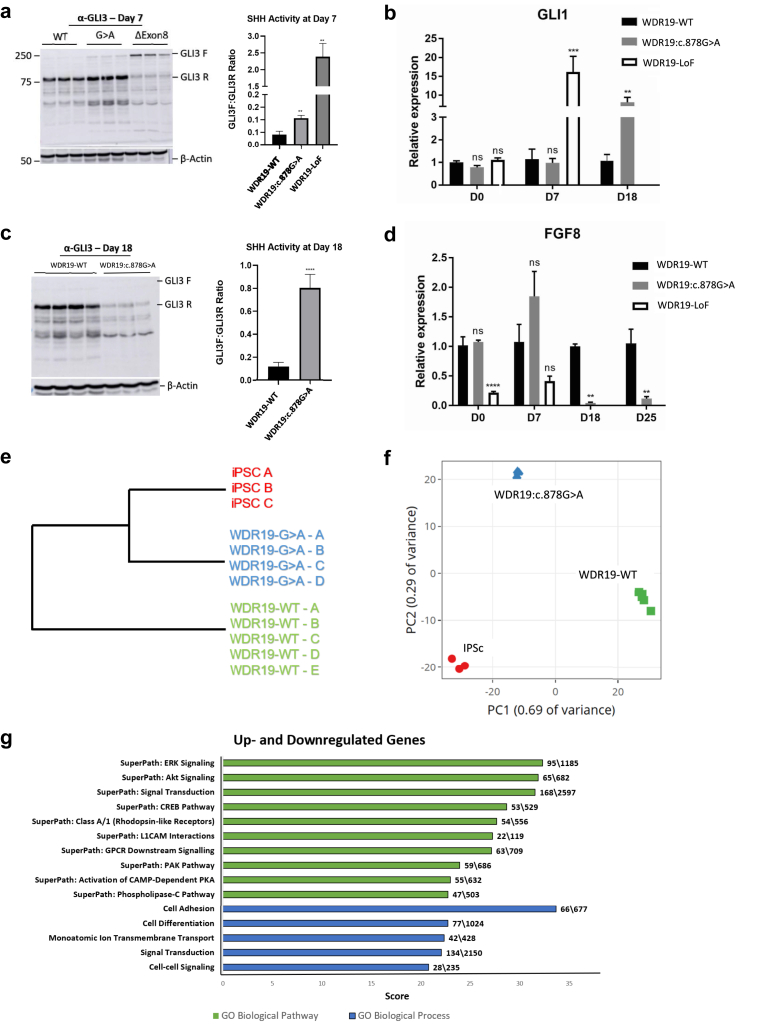
(a) Western blot analysis of GLI3 processing in *WDR19*-WT, *WDR19:C.878G>A*, and *WDR19*-LoF lines on day 7 of differentiation. The GLI3F:GLI3S ratio quantifies Sonic Hedgehog (Shh) pathway activity, showing a positive correlation with pathway activation. (b) GLI1 relative expression during differentiation days 0, 7, and 18 in *WDR19*-WT, *WDR19:C.878G>A*, and *WDR19*-LoF lines. Due to severely disrupted kidney organoid differentiation, *WDR19*-LoF lines were not included beyond the monolayer stage (day 7). (c) Western blot analysis of GLI3 processing in *WDR19*-WT, *WDR19:C.878G>A*, and *WDR19*-LoF lines on day 18 of differentiation. *WDR19*-LoF line was excluded from this analysis due to severely disrupted kidney organoid differentiation. (d) FGF8 relative expression during differentiation days 0, 7, and 18 in *WDR19*-WT, *WDR19:C.878G>A*, and *WDR19*-LoF lines. Due to severely disrupted kidney organoid differentiation, *WDR19*-LoF lines were not included beyond the monolayer stage (day 7). (e) Hierarchical clustering of RNA-sequencing data showing the transcriptional differences between *WDR19* c.878G>A-harboring organoids: *WDR19:C.878G>A* and the *WDR19*-iPSCs lines, and the *WDR19*-WT organoids. (f) Principal component analysis (PCA) plot of bulk RNA sequencing data. The plot illustrates the separation of samples across 3 biological groups: *WDR19*-WT (green squares), *WDR19:C.878G>A* (blue triangles), and *WDR19*-iPSC (red circles). The x-axis (PC1) explains 69% of the variance, whereas the y-axis (PC2) explains 29% of the variance. The clear separation between the *WDR19* mutant lines and the wild-type samples highlights the significant transcriptional differences induced by the pathogenic variant. (g) Top 10 up- and downregulated GO biological pathways (green) and top 5 biological processes (blue) identified by RNA-seq analysis. The analysis is based on 960 genes that were differentially expressed in the same direction (either up- or downregulated) in both hypomorphic WDR19 mutant lines (WDR19:C.878G>A and WDR19-iPSC) compared to WDR19-WT. The numbers on the right indicate the number of altered genes out of the total genes in that GO term. GO, gene ontology; LoF, loss-of function; WT, wild-type.

Considering that the Shh signaling pathway is closely associated with primary cilia function, we conducted an immunoblot analysis of the effector GLI3 protein and transcriptional targets of Shh signaling during the differentiation time course. The hedgehog effector protein GLI3 can exist in a full-length form (GLI3F), which is associated with pathway activation, as well as a shorter, partially processed form (GLI3R), associated with pathway repression. On day 7 of differentiation, the GLI3F:GLI3R ratio was mildly increased in *WDR19:C.878G>A* cells compared with the isogenic control *WDR19*-WT cells. However, in *WDR19*-LoF mutants, the GLI3F:GLI3R ratio was exceptionally high, indicating a significant loss of Shh pathway inhibition (Figure 4a). This suggests that the *WDR19* pathogenic variant disrupts normal Shh pathway regulation in a variant severity dependent manner. To further assess the Shh pathway, the transcriptional target GLI1, a marker of Shh pathway activation, was measured at different time points by real-time polymerase chain reaction. On day 7, GLI1 levels were similar in *WDR19:C.878G>A* missense variant organoids, but approximately 16-folds higher in the nonsense *WDR19*-ΔExon8 cells (Figure 4b). On day 18, exceptionally high activation of Shh was notable even in the *WDR19:C.878G>A* variant, indicating that ciliopathy pathogenesis becomes more prominent as differentiation progresses (Figure 4c). Shh activation was not evaluated in *WDR19*-LoF on day 18 because the cells failed to differentiate to kidney structures, and the morphological differences were too remarkable for comparison. Consistent with these results, *WDR19:C.878G>A* exhibits significantly higher relative GLI1 expression on day 18 (Figure 4b). These findings demonstrate that both hypomorphic and LoF *WDR19* variants result in disinhibited Shh signaling, likely contributing to the disrupted nephron differentiation observed in kidney organoids derived from these mutant lines.

To further characterize the effect of the *WDR19* pathogenic variants on kidney organoids, we performed bulk RNA sequencing on samples from the 3 biological groups: *WDR19*-WT (*n* = 5), *WDR19:C.878G>A* (*n* = 4), and *WDR19*-iPSCs (*n* = 3). Out of 960 differentially expressed genes in the bulk RNA-sequencing (excluding genes shown to be variable across repeated differentiation experiments of a WT iPSC cell line^27^), FGF8, a signaling protein involved in embryonic development, was the most downregulated gene in *WDR19:C.878G>A* compared with *WDR19*-WT, with a fold change of approximately 27 (Supplementary Figure S6). FGF8 is crucial for nephrogenesis, particularly for cell survival, because its absence halts nephrogenesis before reaching the S-shaped body stage.^28^ In mouse embryonic kidney explants, Shh negatively regulates FGF8 expression, which is essential for nephron development and kidney morphogenesis.^29^ Similarly, our kidney organoid model demonstrated a negative correlation between FGF8 expression levels and Shh activation. Specifically, during days 0 to 7 when Shh activity in the *WDR19:C.878G>A* was at most mildly increased, no significant effect on FGF8 levels was observed. In later stages of these days of kidney organoid differentiation (days 18–25) however, FGF8 was significantly low (Figure 4d). In contrast, in the *WDR19*-LoF line, FGF8 was markedly suppressed even in nondifferentiated embryonal cells.

### Gene Expression Analysis Uncovers Dysfunctional Cellular Processes Because of the *WDR19:C.878G>A*; Hypomorphic Variant

For transcriptional profiling, we focused on genes differentially expressed in the same direction (up or down) in both hypomorphic diseased lines as *WDR19*-WT. This approach identified 960 genes as differentially expressed in both pairwise comparisons: *WDR19:C.878G>A* versus *WDR19*-WT and *WDR19*-iPSC versus *WDR19*-WT. Hierarchical clustering revealed greater similarity between the 2 diseased lines, hESC *WDR19:C.878G>A* and the iPSC, compared with the similarity between *WDR19:C.878G>A* and its isogenic control, *WDR19*-WT. This indicates that the effect of the variant was stronger than the cell of origin or any other factor (Figure 4e and f). Gene ontology terms analysis revealed disturbances in cell adhesion, cell differentiation, monoatomic ion transmembrane transport, and cell-cell signaling. These biological processes are critical to kidney development and function, further supporting the observed phenotypic abnormalities in *WDR19*-mutated kidney organoids. Out of the top 4 gene ontology biological pathway terms, 3 were directly related to FGF signaling: ERK/MAPK pathway, Akt Signaling, IP3 Pathway (under CREB Pathway) (Figure 4g). In addition, other gene ontology terms indicate more generalized signal transduction aberrations, including pathways related to G protein-coupled receptor downstream signaling, overall signal transduction, and the phospholipase-C pathway.

### *WDR19* Variants Allow Differentiation Into Cerebral Organoids With Reduced Pathogenicity Compared With Kidney Organoids

**Figure 5:**
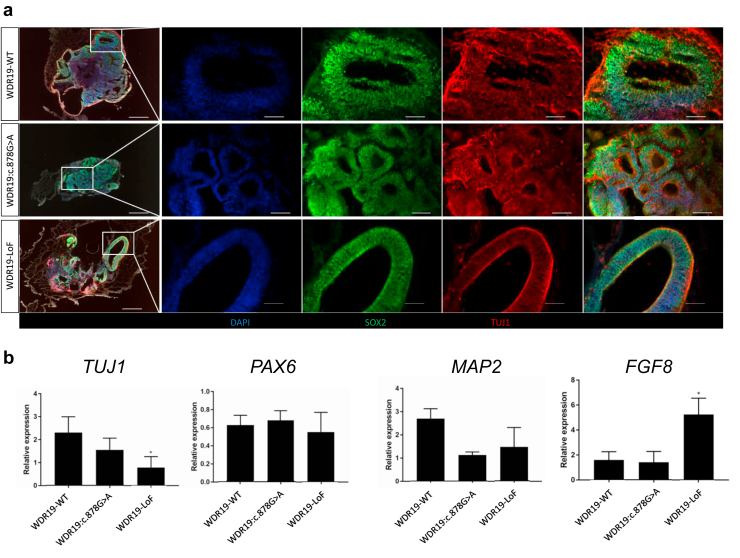
(a) Differentiation of *WDR19*-WT, *WDR19:C.878G>A*, and *WDR19*-LoF cells into cerebral brain organoids. Immunofluorescence staining shows DAPI (blue), SOX2 (green), and TUJ2 (red), in the organoids. All 3 cell lines successfully differentiate into cerebral organoids. (b) Relative expression levels of neuronal markers *TUJ1*, *PAX6*, *MAP2*, and the signaling protein FGF8 on day 77 of differentiation.

To demonstrate the tissue-specific pathogenicity of the *WDR19* variant, particularly given that our patients present with no neurological symptoms, we differentiated *WDR19*-mutated cells into cerebral organoids to assess their differentiation capacity. In contrast to the clear abnormal characteristics observed in kidney organoids derived from *WDR19* mutated lines, differentiation of the *WDR19:C.878G>A* cells to cerebral brain organoids occurred with similar efficiency as WT cells as evidenced by immunostaining of neuronal markers representing neural progenitor cells (SOX2) and early postmitotic neurons (TUJ1) (Figure 5a), and by gene expression (Figure 5b). Furthermore, the *WDR19*-LoF cells were successfully differentiated to cerebral organoids with mild differences in gene expression compared with WT (Figure 5b). These results indicate that there is no significant defect in the differentiation of the mutated cells into cerebral organoids tissue, in contrast to the significant defects observed in kidney organoid differentiation originating from the same cell lines. Remarkably, contrary to the negative correlation between Shh and FGF8 levels observed in kidney organoids, where the LoF variant causes an increase in Shh and suppression of FGF8, cerebral organoids exhibit the opposite relationship, with the LoF variant leading to an increase in FGF8 (Figure 5b). This aligns with previous studies showing tissue-specific differences in these signaling pathways.^30^^,^^31^

## Discussion

The identification of the *WDR19*:c.878G>A variant as a contributing factor to adult-onset, typically nonsyndromic ESKD in the Druze population provides further evidence that the spectrum of ciliopathies is broader than traditionally perceived, extending beyond early-onset and multiorgan disease. Using patient-derived iPSCs and CRISPR-Cas9-edited hESCs differentiated into kidney organoids, we observed that *WDR19*-mutated cells exhibit impaired differentiation into mature nephron structures, characterized by delayed progression at the renal vesicle stage, cystogenesis, and abnormal glomerular and tubular development. Our findings demonstrate that although we identified the *WDR19* variant in an adult-onset ESKD cases, this hypomorphic variant disrupts nephrogenesis, as kidney organoids reflect early kidney development, roughly equivalent to the second trimester.^32^ Naturally, given the pivotal role of cilia in tubular structure function, epithelial cell polarity, and fluid homeostasis, much of the current understanding of *WDR19* variants focuses on tubular abnormalities. However, our study highlights significant glomerular defects in *WDR19*-mutated organoids, which exhibited smaller, less uniform PODXL-positive glomerular structures, and an absence of foot processes, as revealed by electron microscopy—features normally indicative of proper podocyte differentiation. These findings indicate that *WDR19* variants affect the development of both tubular and glomerular structures. Given the glomerular sclerosis, glomerular cysts, and IgA nephropathy–like pathology observed in some of the patients with *WDR19* mutations, it is crucial to expand our understanding of how ciliary dysfunction impacts podocyte function and glomerular integrity in ciliopathies. Of note, unlike many patients with nephronophthisis, who exhibit normal-sized or atrophic kidneys, tubular-specific *WDR19* conditional knockout mice developed significantly enlarged kidneys, closely resembling the phenotype of autosomal dominant polycystic kidney disease.^26^ This discrepancy suggests that the pathogenesis of the disease may extend beyond tubular function alone.

This study is the first to demonstrate that a patient-specific *WDR19* variant causes dysregulation of Shh signaling, a key factor in kidney development, in a human-derived model. We observed an abnormally high GLI3F:GLI3R ratio in *WDR19*-mutated kidney organoids, in both hypomorphic and *WDR19* LoF-mutated organoids, suggesting that proper IFT-A function is essential for Shh pathway inhibition in this patient-specific model. This dysregulation likely contributes to the impaired nephrogenesis and abnormal differentiation observed in the mutant kidney organoids. Furthermore, there was a significant downregulation of FGF8, a critical factor for nephron development, further linking aberrant Shh signaling to the ciliopathy observed in our model. Similar to our findings, studies have shown that FGF8 reduction in the metanephric mesenchyme of mice results in truncated nephrons and a reduced number of renal corpuscles. In addition, complete loss of FGF8 allows cells to reach the renal vesicle stage but prevents further differentiation.^28^^,^^33^ Interestingly, in nonrenal ciliopathy models, upregulation of FGF8 has been linked to craniofacial abnormalities, further highlighting a crucial connection between defective cilia function and dysregulated FGF signaling.^30^^,^^34^

The differentiation of *WDR19:C.878G>A* mutated cells into cerebral organoids did not reveal significant abnormalities. This finding supports the clinical observation that patients with this variant do not exhibit neurological defects. The *WDR19*-LoF organoids exhibit mild abnormalities in neural differentiation compared with the *WDR19*-WT and *WDR19:C.878G>A*. This tissue-specific manifestations suggests that the pathogenic mechanisms driving kidney ciliopathies are distinct from those affecting other tissues, such as the brain. Interestingly, in nonkidney tissues, such as various regions of the central nervous system, reduced GLI3R and enhanced Shh signaling lead to increased FGF8 expression,^30^^,^^31^ indicating a distinct regulatory mechanism compared with kidney tissues, where Shh suppresses FGF8 expression. The differential regulation of Shh and FGF8 signaling between kidney and cerebral organoids underscores the complexity of the molecular pathways involved and highlights the importance of context-dependent gene expression.

In conclusion, this study provides insights into the role of *WDR19* in kidney development and the pathogenesis of ciliopathies in general. Importantly, the use of kidney organoids in this study demonstrates their potential as a valuable tool for modeling early-onset and adult-onset diseases. Furthermore, it allowed validation of the pathogenicity of this rare, significant variant, and showed that *WDR19* variants impact kidney development. Moving forward, investigating how modulating the Shh and FGF8 signaling pathways counteract the effects of *WDR19* variants may potentially offer promising therapeutic possibilities for this genetic kidney disease.

## Disclosure

All the authors declared no competing interests.

## Supplementary Material

Supplementary File (PDF and XLSX) **Supplementary Methods. Figure S1.** Delayed onset of ESKD in *WDR19:c.878G>A* patients compared with a historical cohort. **Figure S2.** Schematic representation of the protocol used for generating kidney organoids. **Figure S3.** The *WDR19* diseased cell lines exhibit normal morphology upon early differentiation stages.Figure S4. Impaired nephrogenic progression in WDR19- G>A kidney organoids is characterized by delayed development at the renal vesicle stage. **Figure S5.** Aberrant ZO1 localization in *WDR19*-mutant kidney organoids. **Figure S6.** FGF8 expression in day 18 kidney organoids as analyzed by RNA-Seq. **Table S1.***WDR19C.878G<A* patients compared with historical cohort table. (XLSX). **Table S2.** List of real-time PCR and PCR primers. **Table S3.** List of antibodies used for Immunostaining and Western Blot. **Table S4.** Composition of media and buffers. **Table S5.** RNA-seq data. (XLSX). **Table S6.** Differentially expressed (DE) genes. (XLSX).

## Footnotes

Supplementary File (PDF and XLSX)

**Supplementary Methods.**
**Figure S1.** Delayed onset of ESKD in *WDR19:c.878G>A* patients compared with a historical cohort. **Figure S2.** Schematic representation of the protocol used for generating kidney organoids. **Figure S3.** The *WDR19* diseased cell lines exhibit normal morphology upon early differentiation stages. **Figure S4.** Impaired nephrogenic progression in WDR19- G>A kidney organoids is characterized by delayed development at the renal vesicle stage. **Figure S5.** Aberrant ZO1 localization in *WDR19*-mutant kidney organoids. **Figure S6.** FGF8 expression in day 18 kidney organoids as analyzed by RNA-Seq. **Table S1.** *WDR19C.878G<A* patients compared with historical cohort table. (XLSX) **Table S2.** List of real-time PCR and PCR primers. **Table S3.** List of antibodies used for Immunostaining and Western Blot. **Table S4.** Composition of media and buffers. **Table S5.** RNA-seq data. (XLSX) **Table S6.** Differentially expressed (DE) genes. (XLSX)
