# Supplementary material for: Elucidating Mechanisms of Hypomorphic WDR19-Related Kidney Failure
Source: Kidney Int Rep. 2025 Jul 24;10(10):3578–91. doi: 10.1016/j.ekir.2025.07.019 (PMC12545897; doi:10.1016/j.ekir.2025.07.019)
Supplement: Supplementary File (PDF and XLSX) — Supplementary Methods. Figure S1. Delayed onset of ESKD in WDR19:c.878G>A patients compared with a historical cohort. Figure S2. Schematic representation of the protocol used for generating kidney organoids. Figure S3. The WDR19 diseased cell lines exhibit normal morphology upon early differentiation stages.Figure S4. Impaired nephrogenic progression in WDR19- G>A kidney organoids is characterized by delayed development at the renal vesicle stage. Figure S5. Aberrant ZO1 localization in WDR19-mutant kidney organoids. Figure S6. FGF8 expression in day 18 kidney organoids as analyzed by RNA-Seq. Table S1.WDR19C.878G<A patients compared with historical cohort table. (XLSX). Table S2. List of real-time PCR and PCR primers. Table S3. List of antibodies used for Immunostaining and Western Blot. Table S4. Composition of media and buffers. Table S5. RNA-seq data. (XLSX). Table S6. Differentially expressed (DE) genes. (XLSX). [file mmc1.pdf]

Supplementary Material – Item S1

Elucidating Mechanisms of Hypomorphic WDR19-Related Kidney Failure

Omer Shlomovitz[1,2,3,7], Yam Ben-Haim[4,7], Netanel Eisenstein[4], Leah Armon[4], Igor Grinberg[5], Sylvie Polak-Charcon[5], Danit Atias-Varon[1,2,3], Guy Chowers[1,2,3], Dror Ben-Ruby[2,3], Achia Urbach[4,8] and Asaf Vivante[1,2,3,6,8]

Supplementary Methods:

Generation of *WDR19:C.878G>A* patients-derived human induced pluripotent stem cells

All procedures were approved by Sheba Medical Center's ethics committee. Patient primary fibroblasts were cultured from a forearm skin biopsy. Fibroblasts were grown in a high-glucose (4.5 g/L) DMEM medium (Sartorius) supplemented with 10% fetal bovine serum (Sigma), 1mM of L-Alanyl-L-Glutamine (Sartorius) and 1% penicillin-streptomycin solution (Sigma). Reprogramming of patient somatic cells into iPSCs was performed using the Epi5™ Episomal iPSCs Reprogramming Kit (Thermo Scientific). Plasmids were electroporated into fibroblasts using the Neon system (Thermo Scientific) according to the following parameters: Pulse voltage 1650 V, pulse width 10 ms, 3 pulses. Following electroporation, the cells were cultured on 6-well plates with fibroblast medium (supplementary Table S4) without antibiotics. All media were supplemented with 3 µM Dot1L inhibitor (Selleckchem) from d1 to d14. On d5, $2x10^6$ MEFs were cultured on 10 cm plate coated with 0.2 % gelatin. The next day, the electroporated fibroblasts were transferred to the cultured MEF plate. On d7 the medium was changed to ESCs medium (supplementary Table S4) and replaced every 2 days. After approximately four weeks, the emerging iPSCs colonies were picked and moved into separate wells for expansion using mTeSR1 medium.

Generation of *WDR19:C.878G>A* patients specific and *WDR19* nonsense ΔExon8 embryonal cells lines

To generate all modified embryonic cell lines, we utilized CRISPR-Cas9 system according to ref. All guide RNAs (gRNAs) were designed using the IDT algorithm and cloned into Cas9 backbone plasmids containing puromycin resistance cassette (Addgene #62988) (PMID: 25430774). For the *WDR19:C.878G>A* ssDNA template design, the mutation site was centrally positioned with flanking sequences, and silent mutations were introduced to create a new restriction enzyme site. CSES7 embryonic stem cells (kindly provided by Prof. Benvenisty, The Hebrew University, Israel) were transfected using Lipofectamine™ Stem Transfection Reagent (Thermo Scientific) according to the manufacturer's instructions with both the gRNA containing plasmid and ssDNA oligo template: 5'-CCATAAAGATAATCTAACCAGCATTGCAGTATCACAGACTCTTAACAAAGTCGCGACATAT GGAGATAACTGgtaagttattttcacatatttttaggaaagcttatatagtaaatactt-3'. The following day, cells were treated with puromycin for 48 h to select successfully transfected clones. Surviving clones were isolated, expanded, and subjected to genomic PCR analysis to confirm the successful generation of knock-in mutation. For the *WDR19* ΔExon8 knockout (KO) cell line a similar approach was used, but with two gRNAs targeting introns downstream and upstream to Exon 8 resulting in the excision of the whole exon, leading to a premature stop codon. Surviving clones were isolated, expanded, and subjected

to genomic PCR analysis to confirm the successful exon excision as seen by the 412 bp shorter band upon electrophoresis, indicating the missing excised sequence of exon 8.

To generate all modified embryonic cell lines, we utilized CRISPR-Cas9 system according to Cong et al.[22]. All guide RNAs (gRNAs) were designed using the IDT algorithm and cloned into Cas9 backbone plasmids containing puromycin resistance cassette (Addgene #62988).[23] For the *WDR19:C.878G>A* ssDNA template design, the mutation site was centrally positioned with flanking sequences, and silent mutations were introduced to create a new restriction enzyme site. CSES7 embryonic stem cells were transfected using Lipofectamine™ Stem Transfection Reagent (Thermo Scientific) according to the manufacturer's instructions with both the gRNA containing plasmid and ssDNA oligo template: 5'-CCATAAAGATAATCTAACCAGCATTGCAGTATCACAGACTCTTAACAAAGTCGCGACATAT GGAGATAACTGgtaagttattttcacatattttaggaaagcttatatagtaaatactt-3'. The following day, cells were treated with puromycin for 48 hours to select successfully transfected clones. Surviving clones were isolated, expanded, and subjected to genomic PCR analysis to confirm the successful generation of knock-in mutation. For the *WDR19*-LoF cell line a similar approach was used, but with two gRNAs targeting introns downstream and upstream to Exon 8 resulting in the excision of the whole exon, leading to a premature stop codon. Surviving clones were isolated, expanded, and subjected to genomic PCR analysis to confirm the successful exon excision as seen by the 412 bp shorter band upon electrophoresis, indicating the missing excised sequence of exon 8.

Directed Differentiation to Kidney Organoids

The differentiation was according to the protocol by Takasato et al. (PMID: 27560173). Undifferentiated induced pluripotent stem cells (iPSCs) and human embryonic stem cells (hESCs) were maintained in feeder-free conditions on Vitronectin (Stemcell Technologies) coated 6-well plates in mTeSR1 medium (Stemcell Technologies). One day before differentiation, the cells were dissociated with TrypLE Select (Thermo Scientific) and 1.4×10^4 cells were plated per well of a 6-well plate on hESC-qualified Geltrex (Thermo Scientific) in 2 mL mTeSR1 medium supplemented with 10 μMY-27632 dihydrochloride (ApexBio). The following morning, cells were treated with 8 μM CHIR99021 for 4 d, followed by FGF9 (200 ng/mL, R&D Systems) and heparin (1 μg/mL, Stemcell Technologies) for 3 d, during which the medium was changed every 1-2 d. On d7, cells were dissociated with TrypLE Select, counted and 250,000 cells were transferred to a ThinCert 0.4 μm pore polyester membrane (Greiner Bio-One). Cells were treated with 5 μM CHIR99021 for 1 h and then incubated in medium containing FGF9 (200 ng/mL) and heparin (1 μg/mL) for 5 d. The culture was continued without added growth factors for up to 24 d, during which medium was changed every two days. Each of the cell lines, *WDR19:C.878G>A*, *WDR19*-LoF, and *WDR19*-iPSCs, was independently differentiated at least three times, representing three biological replicates for each cell line. In each biological replicate, between 15 and 25 separate organoids were generated, which were distributed across 3 to 5 ThinCerts, with 5

organoids per well. In specific designated transwells, Forskolin (Cayman Chemical), an adenyl cyclase activator, was used to induce cyst formation.

Directed differentiation to cerebral organoids

Cerebral organoids were generated as described by Lancaster and Knoblich (PMID: 25188634). In brief, pluripotent stem cells (PSCs) were dissociated into single cells using Accutase (Merck), and approximately 10,000 cells were seeded per well in a 96-well U-bottom plate coated with 30 mg/ml PolyHEMA (Sigma). The cells were cultured in low-bFGF hESC medium supplemented with 50 μM Y-27632 dihydrochloride (Apexbio). After two days, the medium was replaced, and by d4, when the aggregates reached a diameter of 350-600 μm, the medium was changed to remove Y-27632. On d6, the embryoid bodies (EBs) were transferred to a polyHEMA-coated 24-well plate containing 500 μl of Neural Induction Medium. An additional 500 μl of the medium was added after 48h. On d11, the EBs were embedded in Matrigel droplets (BD Biosciences) and cultured in Neurobasal-based differentiation medium. After four days of stationary growth, the droplets were transferred to an orbital shaker (85 rpm.), and the differentiation medium was replaced with one containing B27 with vitamin A (Thermo Scientific). The medium was changed every 3-4 days throughout the remainder of the differentiation process.

Western blot

Total protein was extracted using RIPA buffer. The extracted proteins were separated by 10% acrylamide gel followed by transfer to nitrocellulose membrane. The membranes were blocked with 2% bovine serum albumin in TBST (TBS with 0.05% Tween20). Then, the membranes were incubated with appropriate antibodies (Table S3) and developed using Clarity ECL substrate (Bio-Rad).

Electron microscopy

D24 kidney organoids were initially fixed in 2.5% glutaraldehyde in 0.1M buffered cacodylate for 24h, then in 1% osmium tetroxide for 1 h, following dehydration in a series of increasing ethanol concentrations, and finally embedded in epoxy resin/agar mix (Agar Scientific) for 2 d at 60 °C. Next, semi-thin sections were prepared from the block and relevant areas were selected for ultra-thin sections. These sections were then stained with uranyl acetate and lead citrate. Examination was conducted using a JEOL JEM-1400Flash transmission electron microscope.

Table S2. List of real-time PCR and PCR primers

Gene	Forward	Reverse
GLI1	CCAGCGCCCAGACAGAG	CTGATTGGTGGTGGGGTCAT
FGF8	GACCCCTTCGCAAAGCTCAT	CCGTTGCTCTTGGCGATCA
HPRT	CATTATGCTGAGGATTTGGAAAGG	CTTGAGCACACAGAGGGCTACA
WDR19 E8	TTTAGCCAACACTGACCTGCA	AGGGCCTACTTTGTGTCAGA
WDR19 E9	ATAGGTATGGTGATGGCCGC	ATGGGGTGGAGTGTGCTTAC
WDR19 E8 cDNA	TTCAGCAGGACTTTGGCAAC	CTGTGATACTGCAATGCTGGT
TUJ1	CGCCCAGTATGAGGGAGAT	GGCCTGAAGAGATGTCCAAA
MAP2	CATGGGTCACAGGGCACCTATTC	GGTGGAGAAGGAGGCAGATTAGCTG
PAX6	AGTTCTTCGCAACCTGGCTA	ATTCTCTCCCCCTCCTTCCT

Table S3. List of antibodies used for Immunostaining and Western Blot.

Antibody	Dilution	Host	Supplier	Application
Anti-ECAD 610182	1:300	Mouse	BD Transduction Laboratories™	Immunostaining
Anti-PODXL AF1658	1:500	Goat	R&D Systems	Immunostaining
Anti-alpha Tubulin Ab24610	1:1500	Mouse	Abcam	Immunostaining
Anti-gamma Tubulin Ab179503	1:400	Rabbit	Abcam	Immunostaining
Anti-LIM1/LHX1 Ab229474	1:250	Rabbit	Abcam	Immunostaining
Anti-ZO-1 Ab2272	1:750	Rabbit	Sigma-Aldrich	Immunostaining
Anti-TUJ1 T5076	1:1000	Mouse	Sigma	Immunostaining
Anti-SOX2 Ab5603	1:250	Rabbit	Sigma	Immunostaining
Anti-GLI3 AF3690	1:1000	Goat	R&D Systems	Westernblot
LTL B-1325	1:300		Vector Labs	Immunostaining
Streptavidin DyLight 488 SA-5488-1	1:750		Vector Labs	Immunostaining

Anti-Rabbit IgG H&L Alexa Fluor® 594 Ab150084	1:750	Goat	Abcam	Immunostaining
Anti-Goat IgG H&L Alexa Fluor® 555 Ab150130	1:750	Donkey	Abcam	Immunostaining
Anti-Mouse IgG H&L Alexa Fluor® 647 Ab150111	1:750	Donkey	Abcam	Immunostaining

Table S4. Composition of media and buffers

**Mediums and Buffers**	**Reagents**	**Concentration/Volume**
Fibroblast Medium	DMEM-F12	-
	Fetal Bovine Serum	10%
	Penicillin/Streptomycin	1%
	L-glutamine	1 mM
IPSCs Medium	DMEM/F12	-
	KOSR	10%
	MEM non-essential amino acids	1%
	β-mercaptoethanol	0.1 mM
	Glutamine	1 mM
	Penicillin/Streptomycin	1%
	bFGF	8 ng/ml
Lysis Buffer	Tris (pH=8)	10 mM
	Nonidet P-40	0.45%
	Tween 20	100 µg/ml
	Proteinase K	0.1 mg/ml

Figure S1

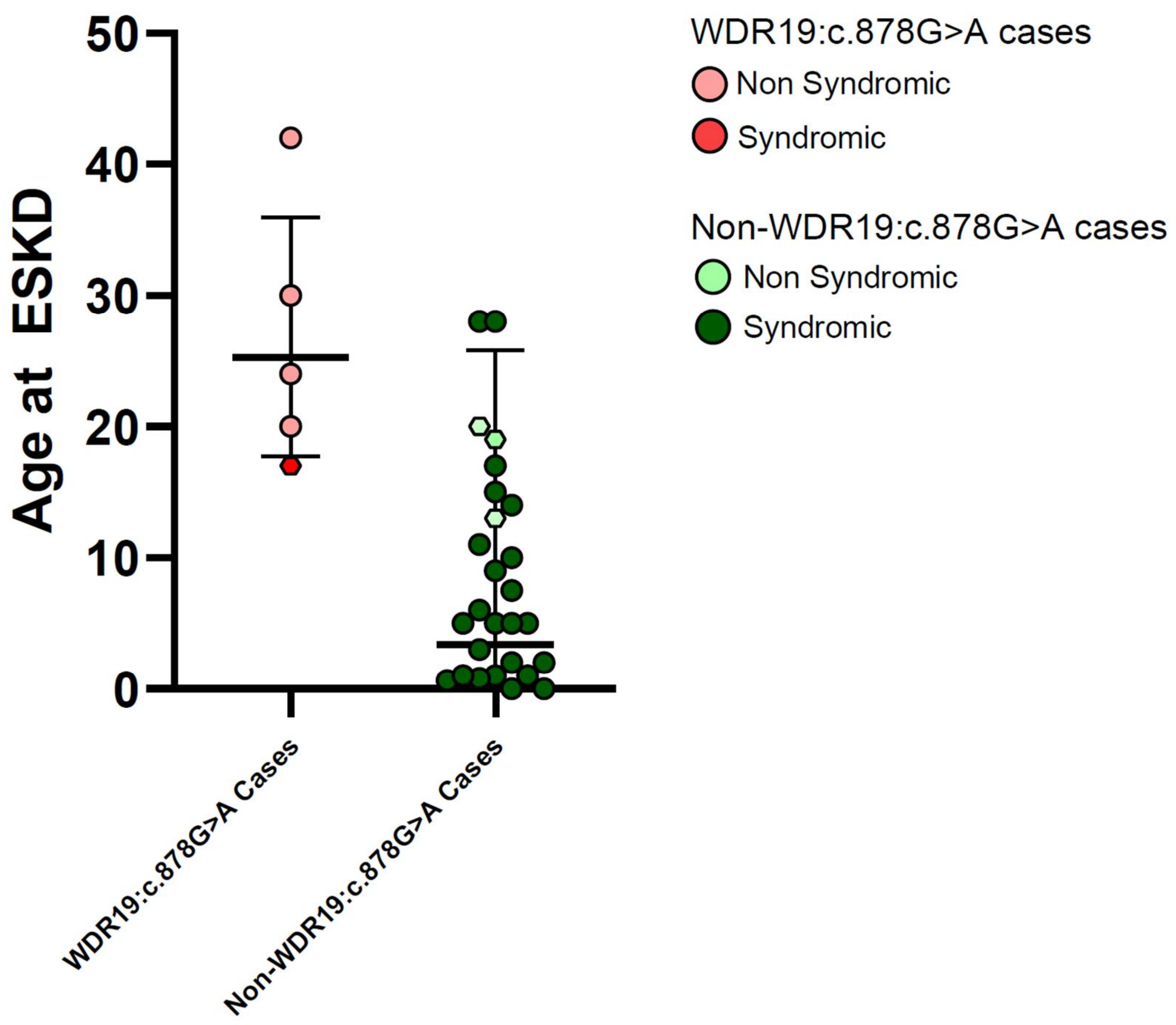

Figure S1. Delayed onset of ESKD in WDR19:c.878G>A patients compared to a historical cohort. Data are presented as scatter plots showing the geometric mean with geometric standard deviation (SD).

Figure S2

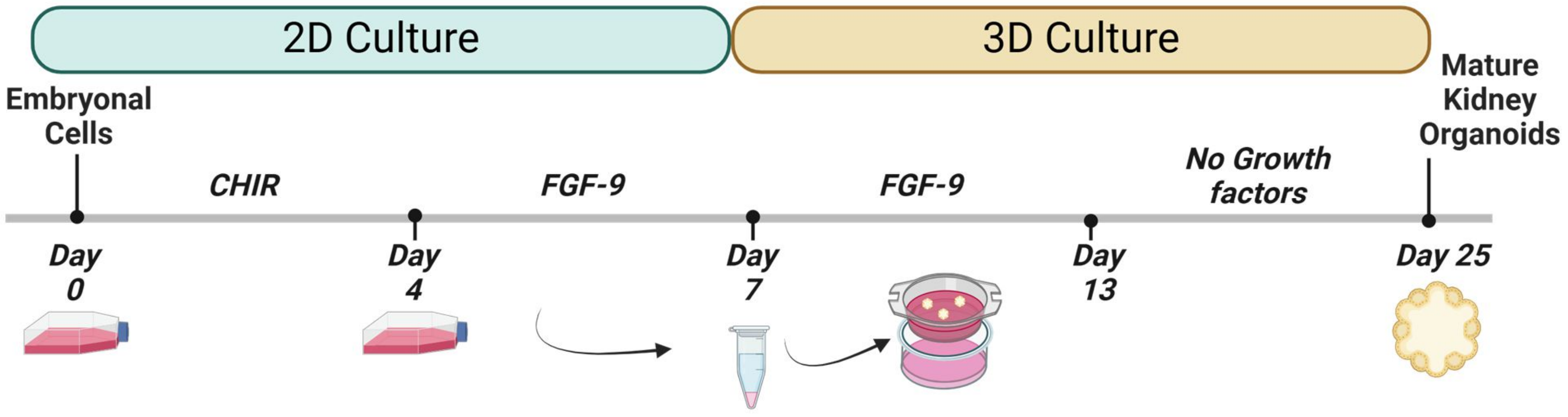

Figure S2. Schematic representation of the protocol used for generating kidney organoids. Cells are cultured as a monolayer for 7 days then transferred as a pellet of 275K cells to a transmembrane system, where they continue to grow at the air-fluid interface. The illustration was created with BioRender.com.

Figure S3

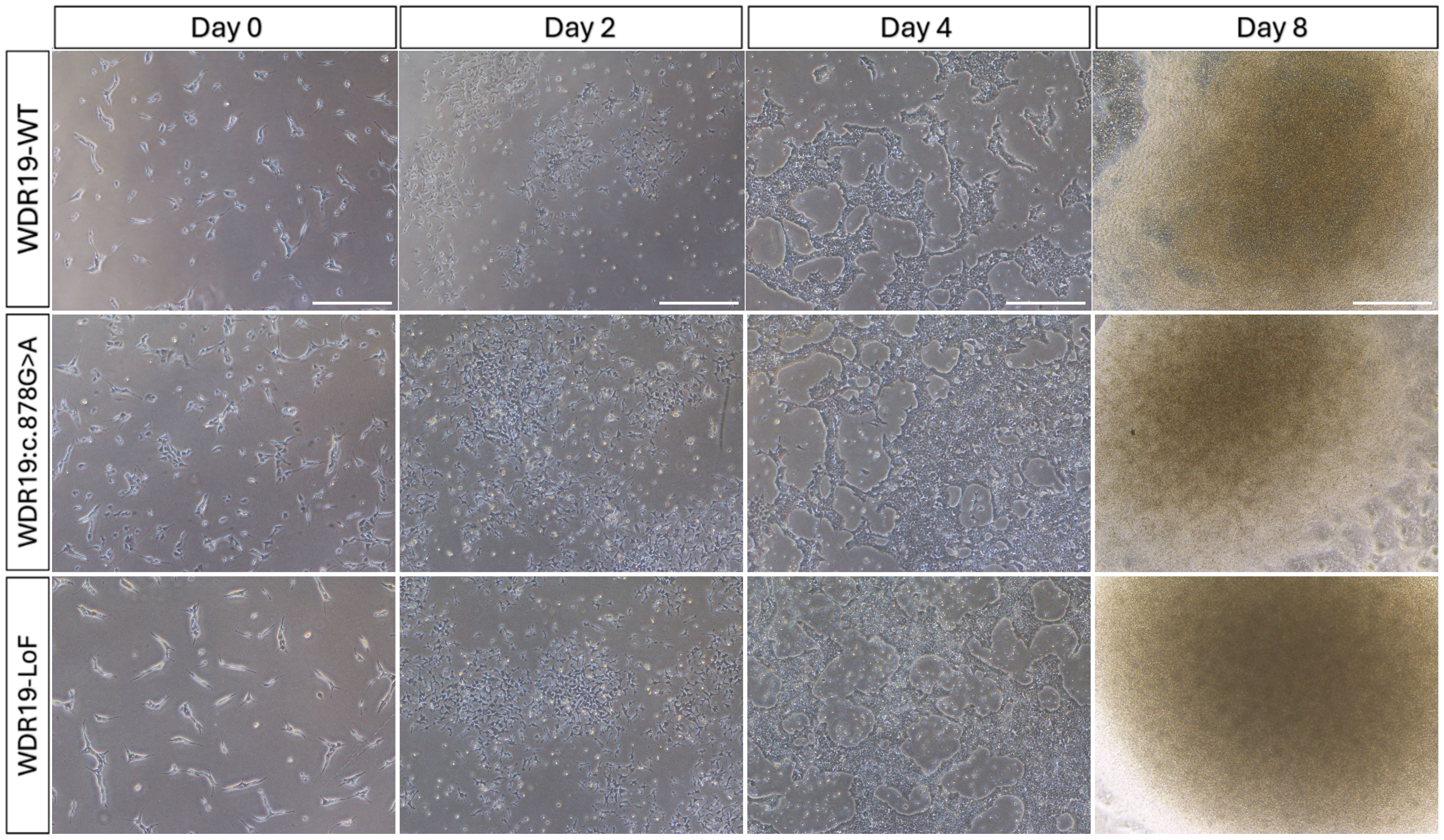

Figure S3. The WDR19 diseased cell lines exhibits normal morphology upon early differentiation stages. Phase contrast images of differentiating cells from day 0 to day 8. Days 0, 2 and 4 are at the monolayer stage whereas day 8 is at the 3D organoid stage. No morphological differences observed between the WDR19-LoF, WDR19:c.878G>A, and WDR19-WT up to day 8 of differentiation. Scale bars: 500 µm.

Figure S4

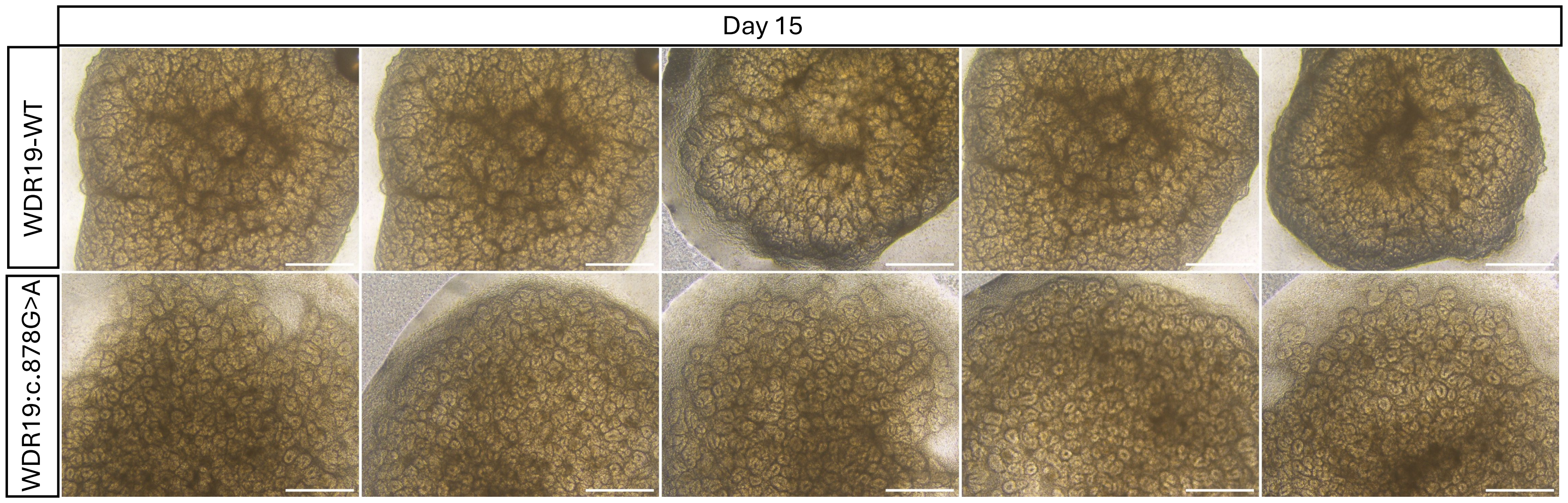

Figure S4. Impaired nephrogenic progression in WDR19-G>A kidney organoids is characterized by delayed development at the renal vesicle stage. Phase contrast images of kidney organoids at day 15 of differentiation. Five different organoids are shown for each line. Scale bars: 500 µm.

Figure S5

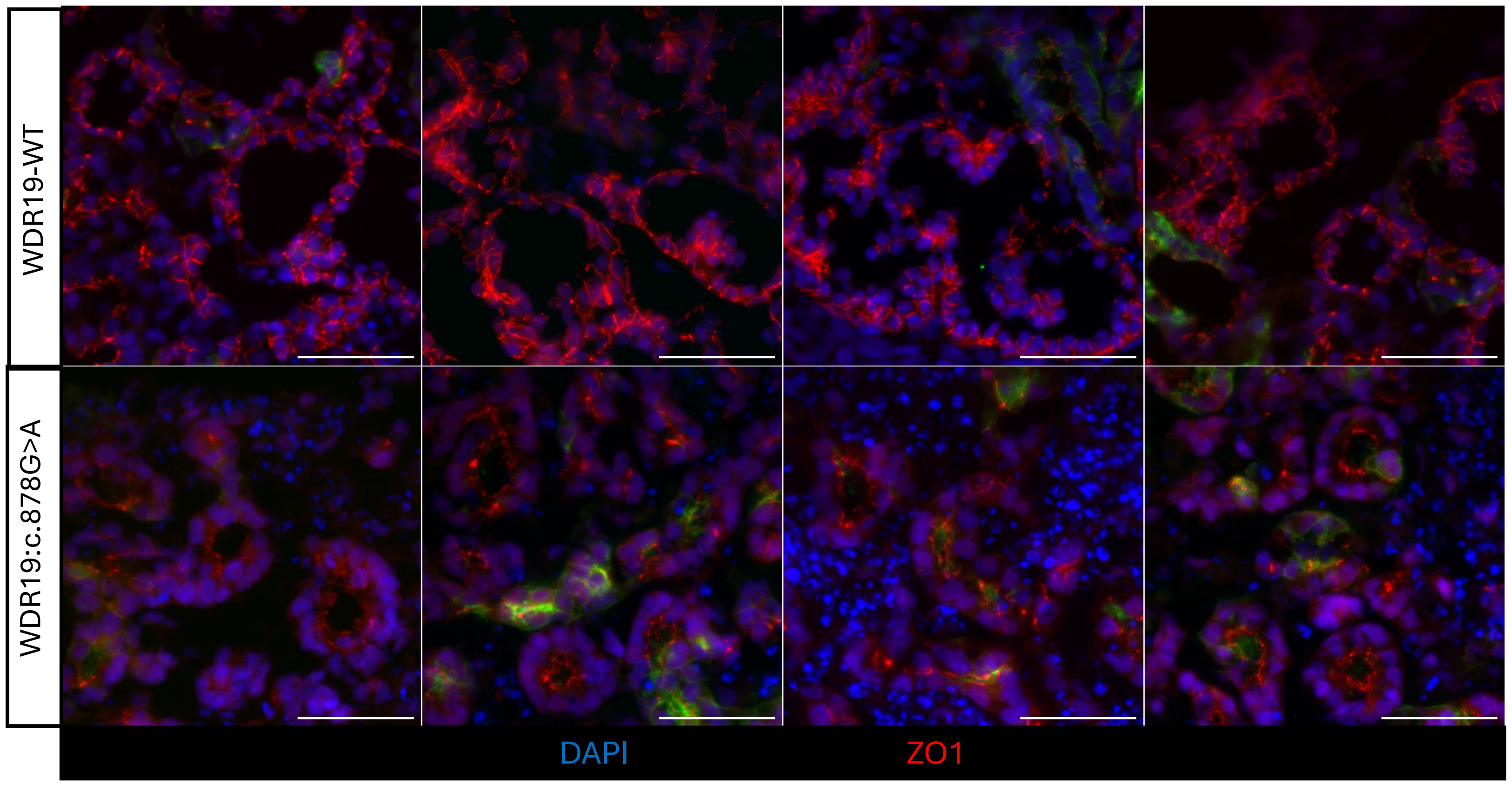

Figure S5. Aberrant ZO1 localization in WDR19-mutant kidney organoids. Immunofluorescence staining for the tight junction protein ZO1 (red), nuclei (DAPI, blue) in day 25 kidney organoids derived from WDR19-WT (top row) and WDR19:c.878G>A mutant (bottom row) lines. Scale bars: 50 µm.

Figure S6

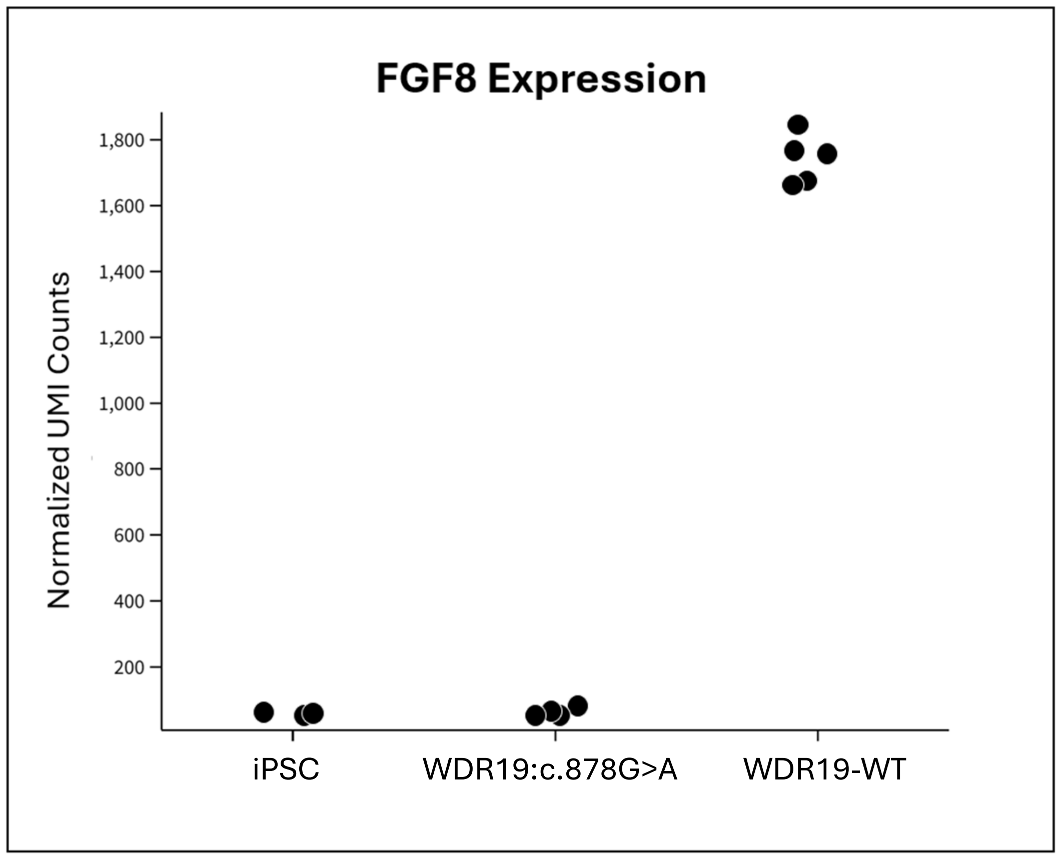

Figure S6. FGF8 expression in day 18 kidney organoids as analyzed by RNA-Seq. Data shown as normalized UMI counts.
